# Kinematic Characteristics and Reliability of Selective Toe Extension Tasks in Young and Older Adults

**DOI:** 10.3390/jfmk11010093

**Published:** 2026-02-26

**Authors:** Seiya Abe, Hitoshi Koda, Takashi Yasuda, Noriyuki Kida

**Affiliations:** 1 Doctoral Programs of Biotechnology, Graduate School of Science and Technology, Kyoto Institute of Technology, Hashigami-cho, Matsugasaki, Sakyo-ku, Kyoto 606-8585, Japan; biwako.ot.abe@gmail.com; 2 Department of Occupational Therapy, Faculty of Rehabilitation, Biwako Professional University of Rehabilitation, 967 Kitasakamachi, Higashiomi 527-8533, Japan; 3 Faculty of Arts and Sciences, Kyoto Institute of Technology, Hashigami-cho, Matsugasaki, Sakyo-ku, Kyoto 606-8585, Japan; kida@kit.ac.jp; 4 Department of Physical Therapy, Faculty of Rehabilitation, Biwako Professional University of Rehabilitation, 967 Kitasakamachi, Higashiomi 527-8533, Japan; t-yasuda@pt-si.aino.ac.jp

**Keywords:** selective toe extension, enslaving phenomenon, intraclass correlation coefficient, aging, inter-toe coupling, ground-contact

## Abstract

**Background**: Toe motor control contributes to postural stability and walking, yet clinical assessments have focused on toe-grip strength. The kinematics of selective toe extension under conditions requiring non-target toes to remain in contact with the ground remain poorly quantified. The aim of the present study was to characterize the kinematics and reliability of selective toe extension tasks using three-dimensional motion capture and to compare young and older adults. **Methods**: A total of 40 participants (20 young adults and 20 older adults) performed three tasks twice: all-toe extension, selective hallux extension, and selective four-toe extension (toes 2–5), with non-target toes required to remain in contact with the ground during selective tasks. Extension angles of the hallux, second, and fifth toes were quantified, and toe-grip strength was measured. Reliability was assessed using the intraclass correlation coefficient (ICC(1,2)). Toe angles were analyzed using two-way analysis of variance (group × condition, including resting and task conditions). **Results**: Toe angles demonstrated moderate to excellent reliability (ICC(1,2) = 0.81–0.95; 95% CI: 0.637–0.974). Compared with all-toe extension, both selective tasks showed reduced extension ranges, indicating an incomplete extension phenomenon in both groups. Significant group × condition interactions were observed for the hallux and second toes. During selective tasks, older adults exhibited greater unintended extension of non-target toes. Toe-grip strength was significantly lower in older adults (*p* < 0.001, Cohen’s d = 2.51). **Conclusions**: Selective toe extension tasks provide reliable kinematic indices of inter-toe motor control by quantifying incomplete extension and associated movements. Older adults showed greater associated movements under ground-contact constraints, suggesting age-related declines in motor coordination and possible reductions in toe flexor strength.

## 1. Introduction

Lower extremity muscle strength is essential for maintaining upright posture and enabling walking. In particular, the hip and knee muscles generate dynamic forces during functional movements such as standing up and squatting, thereby contributing substantially to functional mobility [1]. In contrast, the foot serves as the primary point of contact with the ground, supporting and distributing body weight, providing mechanosensory feedback about external loads, and generating propulsive force, thereby contributing to overall postural stability [2,3]. Furthermore, the toe muscles play a direct role in stabilizing the center of gravity and facilitating forward progression during walking, and their functional importance has been demonstrated.

In older adults, toe muscle strength declines progressively with aging [4], and this reduction has been associated with impaired mobility [5], poorer balance performance, and increased fall risk [6]. In athletes, greater metatarsophalangeal plantarflexion torque has been linked to superior sprinting and jumping performance [7,8]. Taken together, these findings indicate that the toes play a multifaceted and indispensable role in maintaining physical function and optimizing athletic performance across the lifespan.

In recent years, numerous training programs targeting toe function have been developed to enhance balance and prevent falls, with their effectiveness supported by empirical evidence [9,10]. These programs extend beyond simple toe flexion and extension to include a range of exercises, such as towel curls, marble pick-up tasks, toe rock–paper–scissors, maximal toe abduction, and rhythmic toe movements. Their primary objective is to improve toe strength and dexterity, thereby enhancing stability during standing and walking. However, clinical assessments have predominantly focused on toe-grip strength, and few studies have quantitatively examined tasks involving selective motor control of the toes. In particular, complex tasks that require maximal extension of target toes while maintaining contact of non-target toes with the ground lack standardized assessment metrics, and evaluation still relies largely on empirical observation and subjective judgment. Moreover, age-related differences in the ability to perform these tasks remain unclear, and their clinical relevance has yet to be established.

Accordingly, this study aimed to elucidate the kinematic characteristics of a selective toe extension task—in which certain toes remain in contact with the ground while others are maximally extended—using a three-dimensional motion capture system. The analysis focused on the hallux, second, and fifth toes, with extension angles quantitatively assessed. Younger and older adults were compared to explore potential age-related differences in toe motor control.

Although toe function contributes to postural control and gait, tasks performed under weight-bearing conditions involve multiple interacting factors. This complexity makes it difficult to isolate toe-specific motor control mechanisms. Therefore, the seated, non-weight-bearing selective toe extension task used in this study enabled a controlled assessment of toe motor individuation and unintended coupling under a ground-contact requirement while minimizing whole-body balance demands. Establishing the kinematic characteristics and measurement reliability of this task provides an important foundation for future validation under weight-bearing conditions.

## 2. Materials and Methods

### 2.1. Participants

A total of 40 participants were included: 20 healthy young adults (20 males; mean age, 21.0 ± 2.9 years) and 20 community-dwelling older adults (7 males and 13 females; mean age, 82.1 ± 4.9 years). The inclusion criteria for older participants were age ≥ 65 years, independent performance of activities of daily living and the ability to walk safely without assistive devices. Individuals with marked toe deformities, including hallux valgus or claw toe, were excluded; however, no participants met these exclusion criteria. All participants received verbal explanations regarding the study objectives, data usage, and protection of personal information. Written informed consent was obtained prior to participation. The study was approved by the Research Ethics Committee of the Kyoto Institute of Technology (approval No. 2023-97).

### 2.2. Experimental Methods

**Figure 1:**
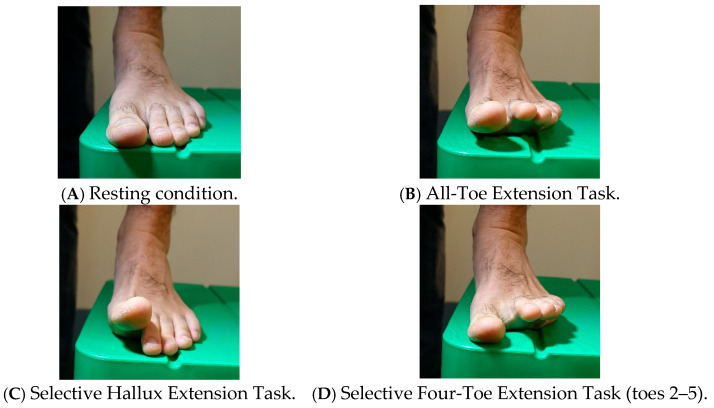
Experimental conditions for toe extension tasks.

Toe angles in the initial foot-flat position were recorded as the resting condition (Figure 1A). Participants then performed three toe extension tasks: the All-Toe Extension Task (Figure 1B), the Selective Hallux Extension Task (Figure 1C), and the Selective Four-Toe Extension Task involving toes 2–5 (Figure 1D). In the All-Toe Extension Task, all five toes were maximally extended. In the Selective Hallux Extension Task, only the hallux was maximally extended, while toes 2–5 remained in contact with the ground. In the Selective Four-Toe Extension Task, toes 2–5 were maximally extended, while the hallux remained in contact with the ground. Each task was performed twice.

All tasks were performed using the dominant foot, defined as the foot the participant would typically use to kick a ball (right, *n* = 36; left, *n* = 4). During testing, participants were seated with the dominant foot placed flat on the floor in the starting position. They maintained an upright trunk posture with their arms crossed over the chest, and both the hip and knee joints were flexed to 90°. The seated position was adopted to minimize fall risk and to ensure participant safety, particularly in older adults. This setup also reduced whole-body balance demands, thereby allowing the assessment to focus specifically on toe motor control and inter-toe coupling. To minimize compensatory hip rotation, a ball approximately 15 cm in diameter was placed between the knees to stabilize the thighs and lower extremities.

Motion data were collected using a three-dimensional motion analysis system (OptiTrack V120 DUO, NaturalPoint Inc., Corvallis, OR, USA). Reflective markers were placed on six anatomical landmarks: the first, second, and fifth metatarsal heads, and the distal phalanges of the hallux, second, and fifth toes. All reflective markers were placed by the same experienced examiner on predefined anatomical landmarks (first, second, and fifth metatarsal heads and corresponding distal phalanges) according to standardized palpation procedures to minimize placement variability.

A right-handed coordinate system was defined relative to the foot, with the mediolateral, anteroposterior, and vertical (upward) directions corresponding to the X-, Y-, and Z-axes, respectively. The extension angle was defined as the angle between the metatarsal head–distal phalanx vector and the Y-axis. For each task, the mean value of two trials was used for analysis.

Comparisons were made across four conditions: the resting condition and the three tasks. Toe extension toward the dorsum of the foot was defined as positive, whereas flexion toward the plantar aspect was defined as negative. Although the foot was placed flat on the floor, small negative angles were occasionally observed at baseline because of marker placement and the natural slight flexion of the toes; this was considered to reflect the anatomical resting posture rather than a systematic measurement error.

In addition, toe-grip strength, a commonly used indicator of toe function, was assessed using a toe-grip dynamometer (Sakai Medical Co., Ltd., Tokyo, Japan). Participants were seated upright with the hip, knee, and ankle flexed at 90°. The grip-bar position was adjusted according to foot length, and participants were instructed to grasp the bar maximally using their toes. Two trials were performed on the dominant foot, and the mean value was used for analysis.

### 2.3. Statistical Analysis

Statistical analyses were performed using IBM SPSS Statistics for Windows, version 30.0 (IBM Corp., Armonk, NY, USA). Measurement reliability for toe extension angles and toe-grip strength was assessed using intraclass correlation coefficients (ICCs). Specifically, ICC(1,1) and ICC(1,2) were calculated to evaluate reliability across repeated trials. Because the mean of two trials was used in subsequent analyses, ICC(1,2) was selected as the primary reliability index and is therefore emphasized in the Results. ICC values were interpreted according to established guidelines [11], with values < 0.50 indicating poor, 0.50–0.75 moderate, 0.75–0.90 good, and >0.90 excellent reliability. Reliability interpretation considered both point estimates and their corresponding 95% confidence intervals.

Toe extension angles of the hallux, second, and fifth toes were analyzed using a two-way mixed-design analysis of variance (ANOVA) with group (young vs. older adults) as the between-subjects factor and condition (resting, all-toe extension, selective hallux extension, and selective four-toe extension) as the within-subjects factor. Group × condition interactions were examined, and partial eta squared (*ηp*^2^) was calculated as an effect size. *ηp*^2^ values were interpreted using conventional benchmarks, with approximately 0.01 indicating a small effect, 0.06 a moderate effect, and 0.14 or greater a large effect. When a significant interaction was detected, simple main effects were tested, followed by Bonferroni-adjusted pairwise comparisons as appropriate. In the absence of a significant interaction, main effects were interpreted using Bonferroni-adjusted pairwise comparisons.

Toe-grip strength was compared between young and older adults using an independent-samples *t*-test. The threshold for statistical significance was set at *p* < 0.05. For kinematic variables showing significant between-group differences, exploratory analyses were conducted within the older adult group to examine potential sex differences.

## 3. Results

### 3.1. Reliability Assessment Using ICCs

Average-measure reliability for toe extension angles was moderate to excellent based on the lower bounds of the 95% confidence intervals (ICC(1,2) = 0.81–0.95; 95% CI: 0.637–0.974; Table 1). ICC(1,2) values ranged from 0.81 to 0.87 for the All-Toe Extension Task, 0.81 to 0.89 for the Selective Hallux Extension Task, and 0.84 to 0.95 for the Selective Four-Toe Extension Task. Toe-grip strength demonstrated excellent reliability (ICC(1,2) = 0.99; 95% CI: 0.986–0.994). The 95% confidence intervals were generally narrow and remained within or close to the moderate-to-excellent reliability range, supporting the stability and precision of these estimates.

### 3.2. Toe Angle

**Figure 2:**
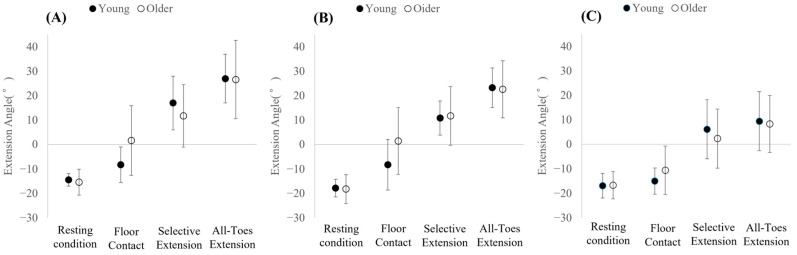
Toe extension angles of the hallux (**A**), second toe (**B**), and fifth toe (**C**) across conditions in young and older adults. Values are presented as mean ± SD.

Toe extension angles of the hallux, second, and fifth toes across the four conditions are illustrated in Figure 2.

#### 3.2.1. Hallux

Mauchly’s test indicated that the assumption of sphericity was not met (W = 0.723, *p* = 0.036); therefore, Greenhouse–Geisser corrections were applied. The two-way mixed-design ANOVA (group × condition) revealed no significant main effect of group (F(1, 38) = 0.10, *p* = 0.749, *ηp*^2^ = 0.003), whereas a significant main effect of condition (F(2.45, 93.00) = 189.25, *p* < 0.001, *ηp*^2^ = 0.833) and a significant group × condition interaction (F(2.45, 93.00) = 5.70, *p* = 0.003, *ηp*^2^ = 0.130) were observed.

To clarify the interaction, simple main effects analyses were conducted. Within each group, hallux extension angles differed significantly across conditions (Bonferroni-adjusted *p* < 0.05). In both groups, hallux extension increased progressively across conditions. The hallux extension angle increased in the following order: resting condition (young adults: −14.5 ± 2.6°, older adults: −15.5 ± 5.3°), Selective Four-Toe Extension Task (young adults: −8.3 ± 7.3°, older adults: 1.6 ± 14.3°), Selective Hallux Extension Task (young adults: 17.0 ± 11.0°, older adults: 11.7 ± 12.8°), and All-Toe Extension Task (young adults: 27.0 ± 10.0°, older adults: 26.6 ± 16.1°).

Between-group comparisons showed that older adults exhibited significantly greater hallux extension than young adults during the Selective Four-Toe Extension Task (F(1, 38) = 7.53, *p* = 0.009, *ηp*^2^ = 0.165), in which the hallux served as a non-target toe required to remain in contact with the ground (young adults: −8.3 ± 7.3°, older adults: 1.6 ± 14.3°).

#### 3.2.2. Second Toe

Mauchly’s test indicated that the assumption of sphericity was not met (W = 0.562, *p* < 0.001); therefore, Greenhouse–Geisser corrections were applied.

The two-way mixed-design ANOVA revealed a significant group × condition interaction for the second-toe extension angle (F(2.137, 81.205) = 4.17, *p* = 0.017, *ηp*^2^ = 0.099).

To further examine this interaction, simple main effects analyses were conducted. Within each group, second-toe extension angles differed significantly across conditions (Bonferroni-adjusted *p* < 0.05). The second-toe extension angle increased in the following order: resting condition (young adults: −17.9 ± 3.6°, older adults: −18.3 ± 5.9°), Selective Hallux Extension Task (young adults: −8.3 ± 10.4°, older adults: 1.4 ± 13.7°), Selective Four-Toe Extension Task (young adults: 10.8 ± 7.0°, older adults: 11.7 ± 12.1°), and All-Toe Extension Task (young adults: 23.3 ± 8.1°, older adults: 22.6 ± 11.7°).

Between-group comparisons for each condition showed that older adults exhibited significantly greater second-toe extension angles than young adults during the Selective Hallux Extension Task (F(1, 38) = 6.41, *p* = 0.016, *ηp*^2^ = 0.144).

#### 3.2.3. Fifth Toe

Mauchly’s test indicated that the assumption of sphericity was not met (W = 0.433, *p* < 0.001); therefore, Greenhouse–Geisser corrections were applied. The two-way mixed-design ANOVA revealed no significant group × condition interaction for the fifth-toe extension angle (F(2.25, 85.31) = 1.92, *p* = 0.148, *ηp*^2^ = 0.048) and no significant main effect of group (F(1, 38) = 0.001, *p* = 0.976, *ηp*^2^ < 0.001). A significant main effect of condition was observed (F(2.25, 85.31) = 104.17, *p* < 0.001, *ηp*^2^ = 0.733). Bonferroni-adjusted post hoc comparisons showed that fifth-toe extension during both the Selective Four-Toe Extension Task and the All-Toe Extension Task was significantly greater than during the resting condition and the Selective Hallux Extension Task (*p* < 0.05). No significant difference was found between the two extension tasks. Additionally, extension during the Selective Hallux Extension Task was significantly greater than during the resting condition (*p* < 0.05).

### 3.3. Toe-Grip Strength

Toe-grip strength was significantly greater in young adults than in older adults (Table 2).

### 3.4. Exploratory Analysis of Sex Differences Within the Older Adult Group

To further examine whether the results may have been influenced by sex distribution within the older adult group, additional exploratory analyses were conducted within the older group (7 males, 13 females).

Because a significant simple main effect between young and older adults was observed for unintended non-target toe movement during the Selective Hallux Extension Task, second-toe extension under this condition was analyzed using an independent-samples *t*-test to examine potential sex differences within the older group.

The independent-samples *t*-test did not reveal a statistically significant difference between older males and females in second-toe extension during the Selective Hallux Extension Task (*t*(18) = −1.64, *p* = 0.119, 95% CI: −23.00 to 2.85, Cohen’s d = −0.77). Given the limited sample size, these exploratory analyses should be interpreted with caution.

## 4. Discussion

### 4.1. Measurement Reliability

Overall, the measurements demonstrated moderate to excellent reliability based on the lower bounds of the 95% confidence intervals. The Selective Four-Toe Extension Task yielded relatively higher ICC values, whereas the All-Toe Extension Task and the Selective Hallux Extension Task showed slightly lower ICC ranges; however, all values remained within the good-to-excellent reliability range. Toe-grip strength also demonstrated very high reliability, consistent with previous reports indicating strong measurement reproducibility [12]. Taken together, these findings suggest that the measurement approach used in this study is stable and reproducible, supporting the use of selective toe extension tasks as reliable assessment tools in both clinical and research settings.

### 4.2. Limited Range of Motion and Associated Movements During Selective Toe Extension

In both the Selective Hallux Extension Task and the Selective Four-Toe Extension Task, the range of motion was more restricted than in the All-Toe Extension Task in both groups. This finding suggests that when certain toes are required to remain in contact with the ground while others are extended, an incomplete extension phenomenon occurs. In addition, small extension angles were observed in toes that were instructed to remain in contact with the ground, indicating involuntary associated movements that appeared to be coupled. These findings support the involvement of the enslaving phenomenon in inter-toe motor control.

The enslaving phenomenon cannot be fully explained by peripheral muscle activation alone and may reflect higher-level neural coordination mechanisms, although the relative contributions of neural and mechanical factors remain unclear. It is characterized by unintentional movements of non-task toes during voluntary movement of a target toe and is believed to arise from anatomical interconnections among muscles and tendons, overlapping peripheral nerve innervation, and shared cortical representations of individual toes within the motor cortex [13,14]. On the dorsum of the foot, the extensor hallucis longus and extensor digitorum longus lie in close proximity and are both innervated by the deep fibular nerve. Therefore, this anatomical and neural proximity may facilitate movement spreading to adjacent toes during selective extension.

Similarly, the flexor hallucis longus and flexor digitorum longus run closely together on the plantar side of the foot and are both innervated by the tibial nerve [15,16]. This anatomical arrangement may promote mechanical coupling between toe flexors, such that increased flexor tension used to maintain ground contact of non-target toes during selective extension tasks could limit extension of target toes, thereby contributing to the incomplete extension phenomenon. In contrast, no significant difference in fifth-toe extension was observed between the Selective Four-Toe Extension Task and the All-Toe Extension Task. The relatively smaller musculature and supporting structures of the fifth toe may partly explain this finding [17,18].

### 4.3. Effects of Aging on Toe Control

In the present study, age-related differences in the extension range of target toes during selective extension tasks were limited, suggesting that the magnitude of the incomplete extension phenomenon was broadly comparable between young and older adults. In contrast, older adults exhibited greater unintended extension of non-target toes under the ground-contact requirement, indicating increased associated movements. This pattern may reflect a reduced ability to suppress unintended movements owing to age-related changes in motor coordination and/or reduced toe flexor strength. However, given the imbalance in sex distribution between groups, the observed differences may also reflect combined influences of age and sex-related factors. Further studies with balanced sampling are warranted to clarify these effects.

Consistent with this interpretation, previous research on finger motor control has reported greater enslaving effects in older adults than in younger adults [14]. Such effects have been interpreted as being associated with age-related changes in neural control and muscle strength, which may influence motor coordination [19,20]. Toe-grip strength was also significantly lower in older adults in the present study. Reduced flexor strength under ground-contact constraints may make it more difficult to maintain toe contact with the ground, thereby facilitating involuntary extension movements.

Taken together, these findings provide clinically meaningful insights into inter-toe coordination under controlled ground-contact conditions. Maintaining ground contact with one toe while extending others appears to require not only muscular strength but also selective motor control. Selective toe extension tasks may therefore serve as a simple dynamic assessment tool for characterizing inter-toe coordination, with potential applications in gait evaluation and fall prevention among older adults.

### 4.4. Limitations of the Study

This study has several limitations. First, only male participants were included in the young adult group, whereas both males and females were included in the older adult group. Although exploratory analyses within the older group focusing on unintended second-toe movement did not reveal statistically significant sex differences, the relatively small number of older male participants may have limited statistical power for these comparisons. Therefore, the independent effects of age and sex cannot be fully disentangled in the present design, and the observed between-group differences should not be interpreted as purely age-related. This sampling imbalance represents a potential confounding factor and warrants cautious interpretation. Future studies with sex-balanced sampling are needed to clarify age- and sex-related influences on toe motor control. In addition, an a priori power calculation was not conducted, as the sample size was determined based on recruitment feasibility. Although significant group × condition interactions were detected for selected variables, the present sample size may have limited sensitivity for detecting smaller interaction effects. Therefore, non-significant interaction findings should be interpreted with caution.

Second, toe extension tasks were assessed only during active movements and only in the dominant foot. Whether similar coordination patterns would be observed during passive movement or in the non-dominant limb remains unclear. Finally, extension angles were derived from marker-based vectors relative to a global coordinate axis. Although standardized procedures and moderate-to-excellent ICC values support acceptable repeatability, minor sensitivity to marker placement and foot alignment cannot be entirely excluded. In addition, because toe motor control was evaluated under seated, non-weight-bearing conditions, its direct relevance to weight-bearing functional activities remains to be established. The clinical implications of these findings require further validation.

## 5. Conclusions

Selective toe extension tasks were characterized by restricted range of motion compared with the All-Toe Extension Task, accompanied by involuntary associated movements in non-target toes under ground-contact constraints. These findings indicate the presence of inter-toe motor coupling, particularly in the hallux and second toes, whereas the fifth toe was relatively less affected. Aging effects were primarily reflected in increased associated movements rather than reduced extension of target toes. Overall, selective toe extension tasks may provide a simple and reliable dynamic assessment of inter-toe coordination and selective motor control, with potential applications in clinical evaluation and fall prevention among older adults.

## Figures and Tables

**Table 1:** Intraclass correlation coefficients for toe extension tasks and toe-grip strength.

Task	Toe	Measurement	ICC	95% CI
All-Toe Extension	Hallux	Single	0.77	0.614–0.874
		Average	0.87	0.761–0.933
	Second	Single	0.76	0.599–0.868
		Average	0.87	0.749–0.929
	Fifth	Single	0.68	0.479–0.819
		Average	0.81	0.648–0.901
Selective Hallux Extension	Hallux	Single	0.81	0.667–0.893
		Average	0.89	0.800–0.944
	Second	Single	0.68	0.467–0.814
		Average	0.81	0.637–0.898
	Fifth	Single	0.74	0.567–0.855
		Average	0.85	0.724–0.922
Selective Four-Toe Extension (Toes 2–5)	Hallux	Single	0.85	0.736–0.918
		Average	0.92	0.848–0.957
	Second	Single	0.73	0.539–0.841
		Average	0.84	0.700–0.915
	Fifth	Single	0.90	0.826–0.948
		Average	0.95	0.905–0.974
Toe-Grip Strength (Dominant Foot)		Single	0.99	0.971–0.992
		Average	0.99	0.986–0.994

ICC(1,1) represents reliability of a single measurement, and ICC(1,2) represents reliability of the average of two measurements. Interpretation of ICC values followed established guidelines (poor <0.50, moderate 0.50–0.75, good 0.75–0.90, excellent >0.90). Notably, in some conditions, the lower bound of the 95% CI fell into a lower reliability category. Abbreviations: CI, confidence interval; ICC, intraclass correlation coefficient.

**Table 2:** Toe-grip strength (kg) in younger and older adults.

	Young (*n* = 20)	Older (*n* = 20)	Cohen’s d	*t*	*p*-Value
Dominant foot	21.0 ± 7.4	6.1 ± 3.9	2.51	7.95	<0.001

Values are presented as mean ± standard deviation. Between-group differences were evaluated using an independent-samples *t*-test.

## Data Availability

The data supporting the findings of this study are available from the corresponding author upon reasonable request.

## Abbreviations

The following abbreviations are used in this manuscript:

ANOVA	Analysis of variance
ICC	Intraclass correlation coefficient
*ηp*^2^	Partial eta squared
